# From detection to elimination: iron-based nanomaterials driving tumor imaging and advanced therapies

**DOI:** 10.3389/fonc.2025.1536779

**Published:** 2025-02-07

**Authors:** Dong Xie, Linglin Sun, Manxiang Wu, Qiang Li

**Affiliations:** ^1^ Department of Radiology, The Affiliated People’s Hospital of Ningbo University, Ningbo, China; ^2^ Department of Radiology, Affiliated Hangzhou First People’s Hospital, Zhejiang University School of Medicine, Hangzhou, China

**Keywords:** iron-based nanomaterials, tumor imaging, magnetic hyperthermia, immunotherapy, artificial intelligence

## Abstract

Iron-based nanomaterials (INMs), due to their particular magnetic property, excellent biocompatibility, and functionality, have been developed into powerful tools in both tumor diagnosis and therapy. We give an overview here on how INMs such as iron oxide nanoparticles, element-doped nanocomposites, and iron-based organic frameworks (MOFs) display versatility for tumor imaging and therapy improvement. In terms of imaging, INMs improve the sensitivity and accuracy of techniques such as magnetic resonance imaging (MRI) and photoacoustic imaging (PAI) and support the development of multimodal imaging platforms. Regarding treatment, INMs play a key role in advanced strategies such as immunotherapy, magnetic hyperthermia, and synergistic combination therapy, which effectively overcome tumor-induced drug resistance and reduce systemic toxicity. The integration of INMs with artificial intelligence (AI) and radiomics further expands its capabilities for precise tumor identification, and treatment optimization, and amplifies treatment monitoring. INMs now link materials science with advanced computing and clinical innovations to enable next-generation cancer diagnostics and therapeutics.

## 1 Introduction

Cancer remains one of the major global health challenges, and mortality rates are expected to continue to increase in the coming decades (1, 2). Nowadays, diagnostic techniques have been improved, but because the biology of cancer is complex, clinical practice still relies on imaging, with about 85% of tumor screenings confirmed by imaging (3, 4). However, traditional imaging techniques such as computed tomography (CT) and magnetic resonance imaging (MRI) often have limitations in terms of specificity and sensitivity. The emergence and development of contrast agents have further enhanced imaging capabilities by improving selectivity, sensitivity, and spatiotemporal resolution (5–7).

In recent years, nanotechnology has emerged as a transformative multidisciplinary field, enabling the exploitation of nanomaterials to address the limitations of conventional imaging and therapy methods (8–10). Among the diverse nanomaterials explored, iron-based nanomaterials (INMs) have gained particular attention due to their distinctive physicochemical properties and versatile biomedical applications (11–17). These materials, including iron oxide nanoparticles (IONPs) (18), element-doped composites (19), and iron-based metal-organic frameworks (MOFs) (20), exhibit unique magnetic properties that make them ideal for both imaging and therapeutic applications. For instance, superparamagnetic iron oxide nanoparticles (SPIONs) are widely utilized as MRI contrast agents to enhance imaging sensitivity and specificity (21), and there is a SPIONs currently approved by the FDA as an imaging contrast agent (22). Additionally, their responsiveness to external magnetic fields enables their application in magnetic hyperthermia for localized tumor treatment (23). The inherent biocompatibility and biodegradability of INMs into metabolizable iron ions further ensure minimal long-term toxicity, distinguishing them from many other nanomaterial platforms (24). Moreover, their surfaces can be functionalized with targeting ligands, therapeutic agents, or imaging markers, enabling applications in multimodal imaging and targeted drug delivery (25).

Recent advances in the design and application of INMs have demonstrated significant potential in cancer diagnosis and therapy. Their integration into imaging techniques such as MRI (26), photoacoustic imaging (PAI) (27), and multimodal platforms (28) have enhanced the sensitivity and precision of tumor detection. Simultaneously, their therapeutic capabilities in photothermal therapy (PTT) (29), immunotherapy (30), and magnetic hyperthermia (31) have shown promising preclinical outcomes. Moreover, the incorporation of these materials with emerging technologies like artificial intelligence (AI) and radiomics offers new opportunities for data-driven diagnosis and optimized treatment strategies, underscoring their potential to revolutionize personalized cancer therapy (32).

**Scheme 1:**
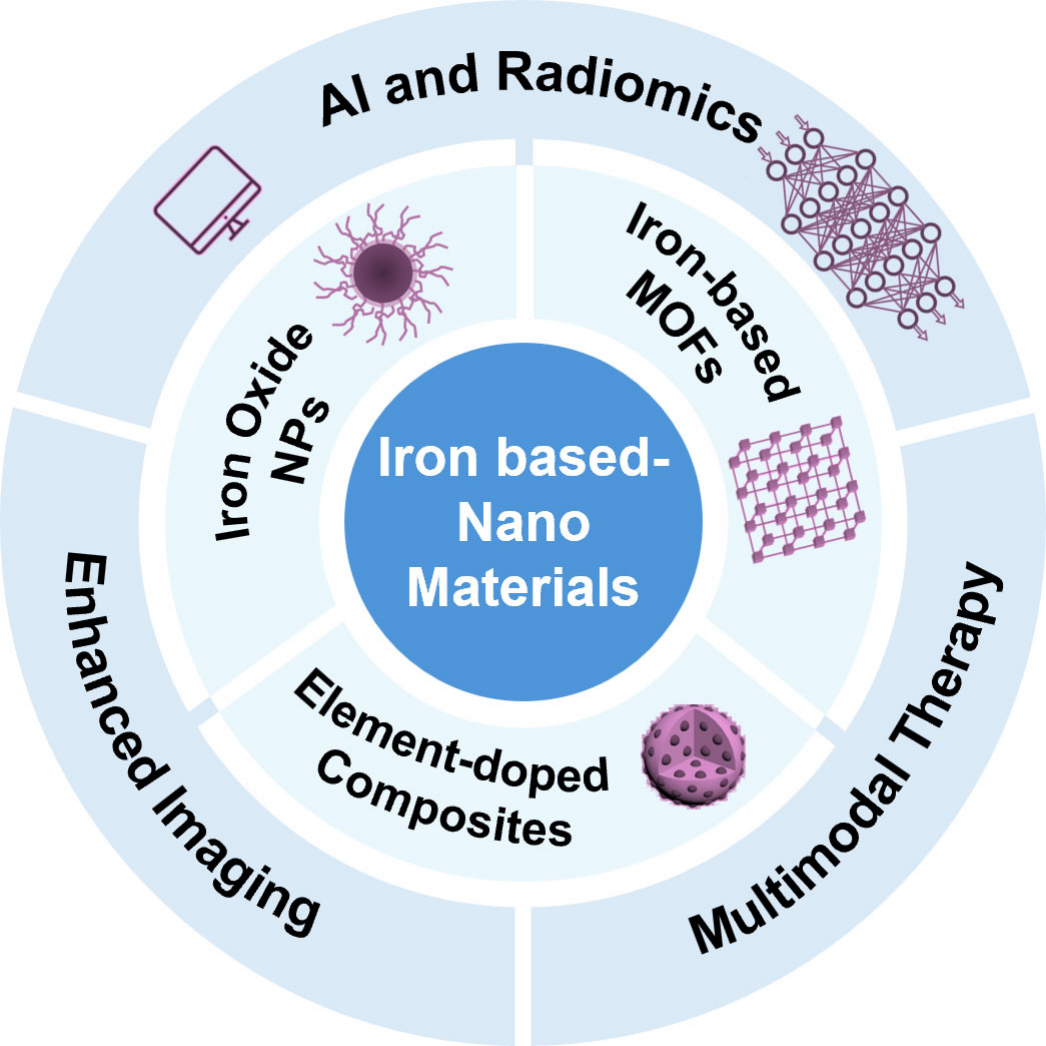
The categorization of iron-based nanomaterials as enhanced materials for tumor Imaging and therapy, and their potential use combined with AI and radiomics.

This review provides a comprehensive overview of the recent progress in INMs for tumor imaging and multimodal therapy (**Scheme 1**). It highlights their contributions to enhancing imaging sensitivity and specificity through advanced molecular imaging techniques and their therapeutic applications in multimodal therapy such as immunotherapy, and magnetic hyperthermia. Furthermore, we explore their integration with computational tools such as AI and radiomics to facilitate precise diagnosis, predictive modeling, and personalized treatment strategies. By bridging the gap between innovative material science and clinical oncology, this review aims to elucidate the transformative potential of INMs in addressing unmet clinical needs and shaping the future of personalized cancer medicine.

## 2 The structure and versatility of iron-based nanomaterials

INMs have emerged as versatile platforms for cancer diagnosis and therapy due to their unique physicochemical properties and diverse functionalization capabilities (33). These materials contain the following three major classes: IONPs (34, 35), iron-doped nanocomposites (36–38), and iron-based MOFs (39–41), and each category exhibits distinct characteristics that make them suitable for various biomedical applications (42), particularly in tumor imaging and multimodal therapies. Each category of INMs offers unique advantages tailored to specific biomedical applications (**Table 1**). IONPs are highly developed for imaging applications (50) due to their strong magnetic properties and clinical safety profile. Besides, element-doped iron nanocomposites provide enhanced multifunctionality (51), enabling simultaneous imaging and therapy, while iron-based MOFs excel in drug delivery and light-responsive therapeutic applications (52). However, challenges remain in optimizing their biocompatibility, ensuring consistent large-scale synthesis, and addressing regulatory barriers for clinical translation.

**Table 1:** Summary of the key features and biomedical applications of INMs.

Material	Key Features	Applications	Reference
IONPS	Superparamagnetic, biocompatible	MRI, magnetic hyperthermia, targeted drug delivery	(34, 35, 43, 44)
Element-Doped Nanocomposites	Enhanced magnetic & optical properties	MRI, multimodal imaging, CDT, PTT	(36–38, 45–47)
Iron-based MOFs	High porosity, tunable structure	Drug delivery, PTT, PDT, combination therapies	(39–41, 48, 49)

### 2.1 Magnetic excellence in iron oxide nanoparticles

Iron oxide nanoparticles, particularly those composed of magnetite (Fe_3_O_4_) and maghemite (γ-Fe_2_O_3_), are the most widely studied INMs due to their unique superparamagnetic properties. These properties arise from the ability of individual nanoparticles to exhibit magnetism only in the presence of an external magnetic field, making them highly effective as contrast agents in MRI. Their superparamagnetic also facilitates their use in magnetic hyperthermia, where alternating magnetic fields (AMF) induce localized heating to target tumor cells selectively (53). In addition to their magnetic properties, IONPs are biocompatible and biodegradable, breaking down into iron ions that are naturally metabolized in physiological pathways such as hemoglobin synthesis (54). This inherent biodegradability ensures minimal long-term toxicity (55), which is a critical consideration for clinical applications. Furthermore, the surface of c can be functionalized with various biomolecules, such as polyethylene glycol (PEG), antibodies, or small-molecule drugs, enabling targeted delivery and reducing off-target effects (43).

Recent advancements in synthetic methods, such as co-precipitation, thermal decomposition, and hydrothermal techniques, have enabled precise control over the size, shape, and magnetic properties of IONPs, further enhancing their performance in imaging and therapy (56). For instance, smaller nanoparticles (<20 nm) demonstrate enhanced pharmacokinetics and tumor penetration (57), while larger particles provide stronger magnetic responses for imaging applications (58).

**Figure 1:**
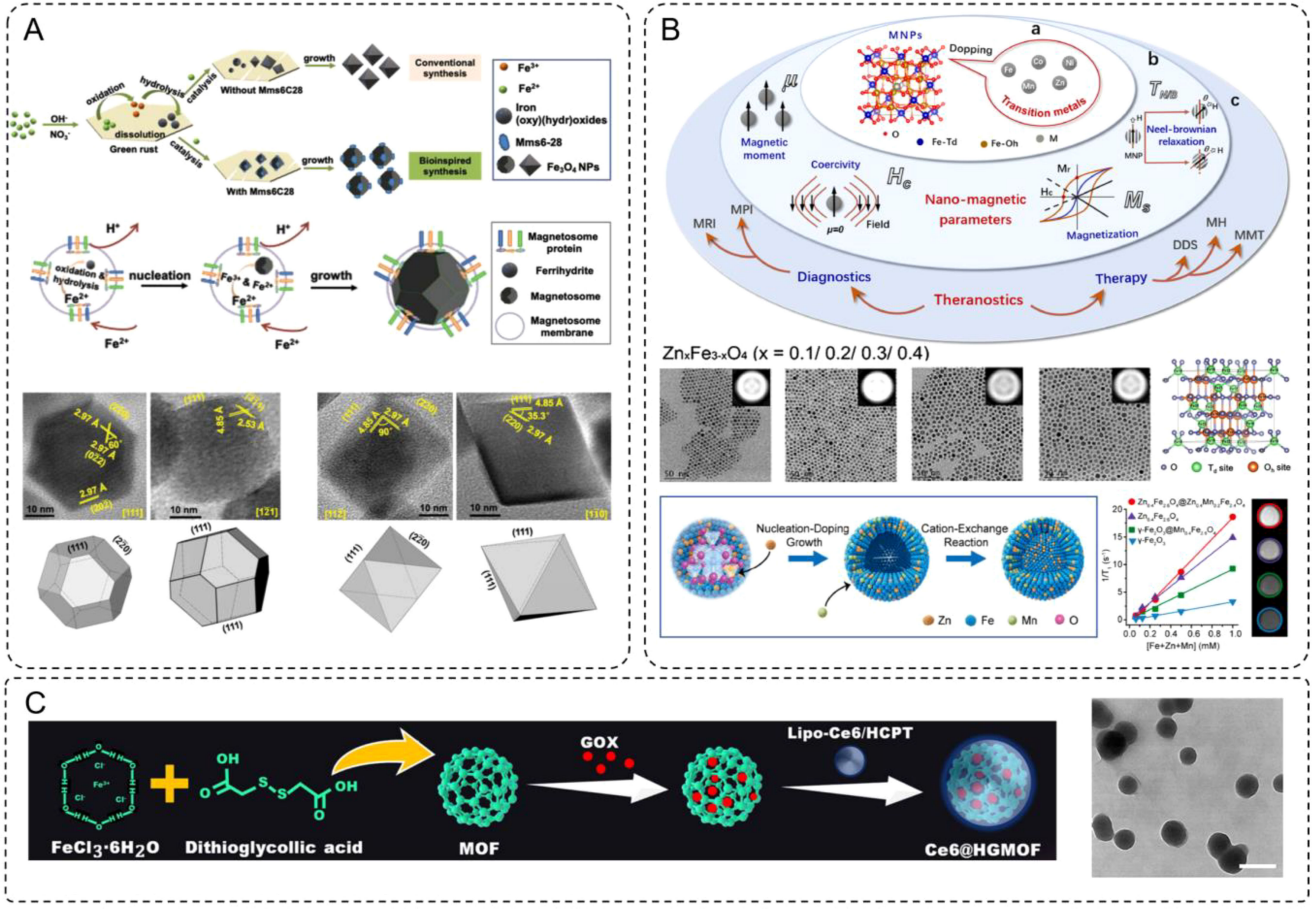
**(A)** The formation scheme of Fe_3_O_4_ NPs through conventional and bioinspired synthesis, and biosynthesis. HRTEM images and three-dimensional morphologies from different zone axes of the IONPs prepared in the presence. **(B)** Transition metal-doped magnetic nanoparticles (TMNPs) for biomedical applications. The doping mechanism of ferrite with different amounts of zinc doping in the hydrophobic phase and the variation of its total magnetic moment. **(C)** Synthetic steps and TEM for Ce6@HGMOF (scale bar: 200 nm).

The work by Mao et al. provided an exemplary case of bioinspired synthesis for magnetosome-like Fe_3_O_4_ nanoparticles (IONPs), aligning with the discussed characteristics of IONPs in terms of magnetic properties, size control, and biocompatibility (44). Using a synthetic peptide, Mms6-28, which mimics the structure and function of the magnetosome membrane protein Mms6, the authors achieved the precise fabrication of Fe_3_O_4_ nanoparticles with uniform cubo-octahedral morphology under partial oxidation conditions (**Figure 1A**). The polypeptide played a dual role by binding iron ions to initiate nucleation and selectively attaching them to the [100] and [111] crystal planes, thereby suppressing the formation of high-index facets. This process allowed us to produce nanoparticles with controlled sizes ranging from approximately 33.4 nm to 24.2 nm as the concentration increased. The resultant particles exhibited superior magnetic properties, including a saturation magnetization of 69.8 emu/g, outperforming conventional co-precipitation methods.

Ferumoxytol, an FDA-approved iron-based MRI contrast agent, exemplifies the clinical translation of IONPs into medical applications (59). With its excellent safety profile and unique pharmacokinetics, ferumoxytol has reshaped contrast-enhanced MRI practices. Unlike gadolinium-based agents, ferumoxytol provides extended imaging windows and avoids nephrotoxicity, making it particularly suitable for patients with chronic kidney disease (60, 61). Clinical trials have demonstrated ferumoxytol’s utility across a range of applications, including vascular imaging, inflammation assessment, and tumor characterization (62). While ferumoxytol represents a significant step toward the clinical use of IONPs, challenges persist. Large-scale synthesis, ensuring biocompatibility, and regulatory hurdles remain key barriers (63). The ongoing development of iron-based contrast agents, such as those featuring specific modifications, represents a promising avenue for next-generation imaging solutions. However, achieving broader clinical impact will require overcoming obstacles such as data standardization for imaging interpretation, validation of model performance across diverse clinical scenarios, and integration into multimodal imaging platforms.

### 2.2 Enhanced performance through elements doping

Element-doped iron-based nanostructures represent an advanced class of materials where non-iron elements, such as manganese (Mn), bismuth (Bi), or gadolinium (Gd), are incorporated directly into the iron-based matrix. This doping strategy significantly enhances the inherent magnetic and catalytic properties of iron while introducing additional functionalities, such as improved redox activity or optical characteristics. For instance, Mn-doped IONPs exhibit increased saturation magnetization for enhanced magnetic hyperthermia (64), while Bi-doped nanoparticles offer dual MRI-CT imaging capabilities due to their magnetic and X-ray attenuation properties (65).

These doped nanostructures have gained attention for their potential in multimodal cancer diagnosis and therapy. By improving Fenton reaction efficiency, dopants such as Co or Mn optimize reactive oxygen species (ROS) generation for chemodynamic therapy (CDT) (45), while elements like Zn or rare-earth metals impart fluorescence or photothermal properties for optical imaging and PTT (46). This versatility makes element-doped iron-based nanostructures highly promising candidates for integrated diagnostic and therapeutic applications.

The study by Du et al. provides a comprehensive demonstration of how transition metal ion doping can precisely tune the properties of iron-based nanostructures for enhanced biomedical applications, aligning closely with the concept of element-doped INMs (47). By substituting divalent transition metal ions such as Mn^2+^, Co^2+^, Zn^2+^, and Ni^2+^ into ferrite nanoparticles, the researchers achieved significant enhancements in magnetic properties (**Figure 1B**), including increased saturation magnetization (Ms), improved magnetic anisotropy (K), and optimized relaxation times for MRI. The doping process involves cation exchange in the spinel crystal lattice, where the dopants occupy tetrahedral and octahedral sites, selectively altering the magnetic dipole alignment and the overall magnetic moment of the nanostructures.

One notable example is the Zn-doped ferrite system (Zn_x_Fe_3-x_O_4_), where a controlled doping level (x = 0.2) achieved maximum saturation magnetization (66.8 emu/g). The authors demonstrated that Zn ions preferentially occupy tetrahedral sites, replacing Fe^3+^ and partially converting Fe^2+^ to Fe^3+^ at octahedral sites to maintain charge balance. This reorganization results in enhanced magnetic properties and biocompatibility, making these nanoparticles highly effective for MRI contrast enhancement and targeted imaging. Beyond individual dopants, the synergistic effects of dual-metal doping were also explored. For example, Zn_x_Mn_y_Fe_3-x-y_O_4_ nanoparticles demonstrated enhanced T_1_-weighted imaging for MRI, with r_1_ relaxivity values up to 22.2 mM^-1^s^-1^, significantly outperforming clinically used gadolinium-based contrast agents. These nanoparticles also exhibited superior magnetothermal conversion efficiency for magnetic hyperthermia, with specific loss power (SLP) values reaching 432 W/g under optimal conditions, enabling efficient cancer cell ablation.

### 2.3 Iron-based metal-organic frameworks for multifunctional solutions

Iron-based MOFs are porous, crystalline materials constructed from iron ions or clusters coordinated with organic linkers. These materials exhibit high surface area, tunable porosity, and the ability to encapsulate or adsorb therapeutic agents, making them promising candidates for drug delivery and imaging applications (66). The modular nature of MOFs allows for precise tuning of their physicochemical properties (67). For instance, the incorporation of iron ions enhances their magnetic properties, enabling their use as MRI contrast agents (68, 69). Additionally, their high porosity facilitates the loading of chemotherapeutic agents, photosensitizers, or imaging probes, enabling simultaneous drug delivery and imaging. Iron-based MOFs have also shown potential in photothermal and photodynamic therapy (48) due to their ability to generate localized heat or ROS upon light irradiation.

As an example of recent advancements, Chen et al. developed an iron-based MOF system (Ce6@HGMOF) that integrates a photosensitizer (Ce6), glucose oxidase (GOX), and a chemotherapeutic drug (HCPT) (49). This multifunctional platform addresses tumor hypoxia, a key limitation in PDT efficacy, by catalyzing the conversion of H_2_O_2_ into oxygen through its catalase-like activity, thereby alleviating the hypoxic tumor microenvironment (**Figure 1C**). Additionally, GOX-mediated glucose depletion enhances “starvation therapy”, while the generation of hydroxyl ·OH through the Fenton reaction induces ferroptosis. The synergistic combination of PDT, ferroptosis, and starvation therapy results in significant anti-tumor effects both *in vitro* and *in vivo*, demonstrating the potential of iron-based MOFs for multifunctional cancer therapies. It underscored the versatility of iron-based MOFs in designing intelligent delivery systems for tumor imaging and therapy, showcased their adaptability for advanced cancer treatment strategies. The integration of such materials with emerging technologies, such as AI-assisted analysis and radiomics, offers promising opportunities for personalized therapy planning and optimization.

## 3 Advancing tumor imaging with iron-based nanomaterials

INMs have demonstrated significant potential in enhancing tumor imaging techniques due to their unique magnetic, optical, and functional properties. Among the many applications, MRI and PAI, along with multimodal imaging, are the most extensively studied, showcasing their ability to improve sensitivity, specificity, and diagnostic accuracy (70–72).

### 3.1 Magnetic resonance imaging

MRI is a widely used non-invasive diagnostic tool in oncology, offering high spatial resolution and detailed anatomical and functional imaging (73). However, its application in detecting small or early-stage tumors is often limited by low sensitivity, creating a demand for contrast agents that can enhance imaging quality (74). INMs, particularly SPIONs, have shown great promise in addressing these challenges (75). By enhancing the contrast in T_1_- or T_2_-weighted images, these materials have significantly advanced the capabilities of MRI in tumor detection and characterization (76).

**Figure 2:**
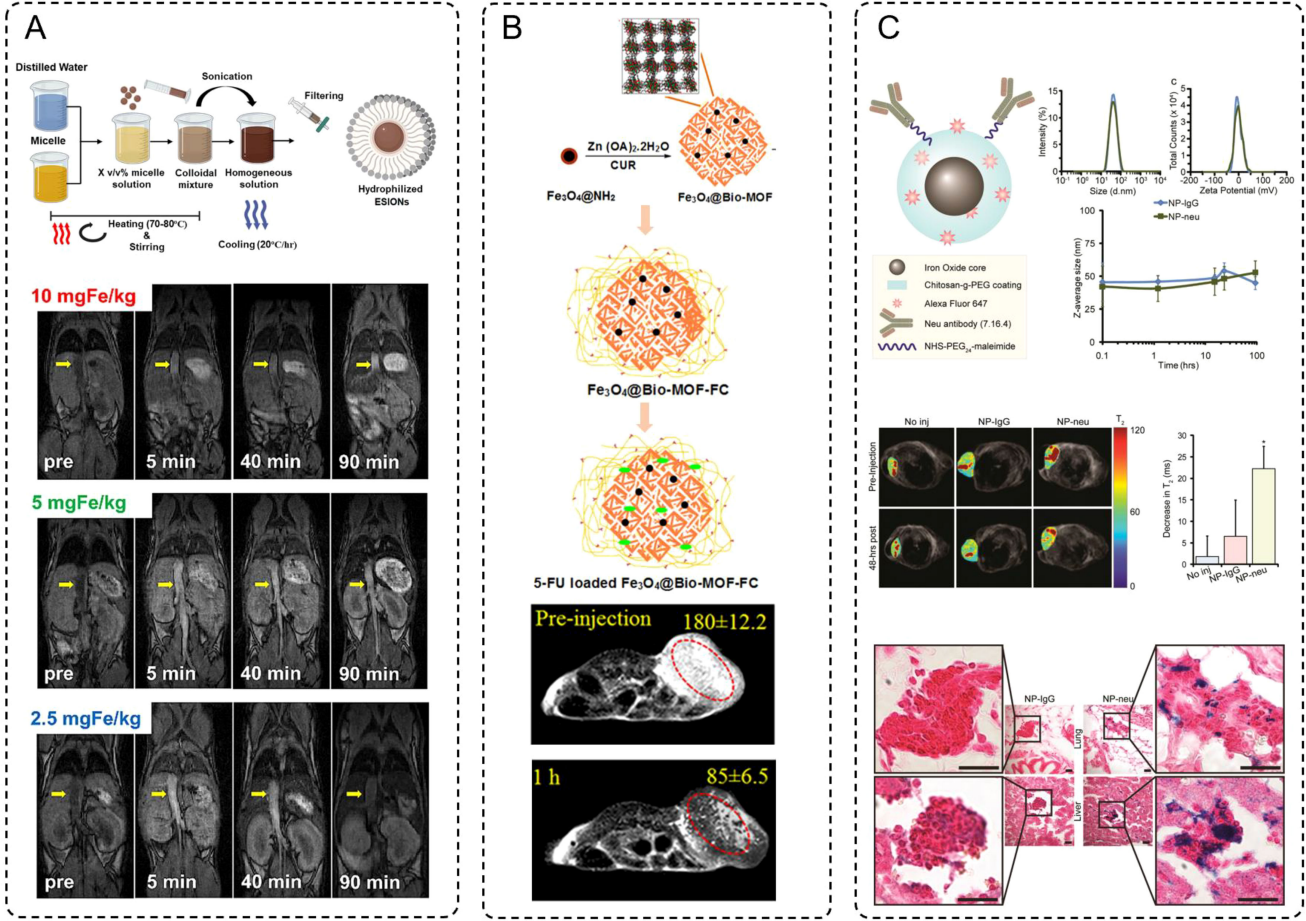
**(A)** Schematic procedure of hydrophilic conversion using micelle encapsulation method, and the MRI signal compared between 3 different concentrations of ESIONs. **(B)** Sequential steps for the preparation of 5-FU loaded Fe3O4@Bio-MOF-FC. And *in vivo* T2-weighted MRI images of tumor bearing BALB/c mice before injection and 10 min or 1 h after injection of Fe3O4@Bio-MOF-FC NCs. **(C)** NP architecture and characterizations, and MRI of NP treated transgenic breast cancer mice. Futher prussian blue stained histology images of micrometastases in lungs and livers from NP-IgG (control) and NP-neu (targeted) treated transgenic breast cancer mice. Scale bars correspond to 20 μm.

The imaging enhancement provided by IONPs stems from their unique magnetic properties, which influence the relaxation times of protons in surrounding tissues. These nanoparticles can act as either T_1_ (positive) or T_2_ (negative) contrast agents depending on their size, surface charge, and magnetic characteristics. Smaller nanoparticles, characterized by high surface relaxivity, are often optimized for T_1_-weighted imaging due to their ability to shorten longitudinal relaxation time, thereby brightening the image (77). For example, the study by Suh et al. provided evidence to support the advantages of extremely small-sized iron oxide nanoparticles (ESIONs) for T_1_-weighted MRI contrast (78). Using a scalable micelle encapsulation method, the researchers achieved a hydrophilic coating of 3 nm ESIONs, resulting in nanoparticles with a uniform hydrodynamic size of 9.35 nm and remarkable stability in physiological environments. (**Figure 2A**) This ultrasmall size offered significant benefits, including reduced uptake by the reticuloendothelial system, which prolonged their circulation time in the bloodstream and enhanced metabolic clearance via the hepatobiliary pathway, minimizing long-term accumulation and renal excretion. Mechanistically, the size reduction also optimized their relaxivity properties, achieving high longitudinal relaxivity (r_1_) and a favorable r_2_/r_1_ ratio, which are critical for effective T_1_-weighted imaging. MRI studies further demonstrated that these ESIONs produced strong T_1_ contrast enhancement at low to moderate concentrations, with reduced risks of signal quenching often associated with larger IONPs. Collectively, these features highlight the dual benefits of size optimization, improving both the imaging performance and the biocompatibility of ESIONs, making them highly promising for precise tumor imaging and other diagnostic applications.

In contrast, larger nanoparticles induce stronger magnetic field distortions, making them ideal for T_2_-weighted imaging, where they enhance contrast by producing signal darkening. Especially, iron-based MOFs with a larger size have significantly enriched the capabilities of T_2_-weighted MRI imaging. Among these, the study by Nejadshafiee et al. highlights the development of a magnetic bio-metal-organic framework (Fe_3_O_4_@Bio-MOF) coated with a folic acid–chitosan conjugate, showcasing its potential as a theranostic agent (79). This material leverages its mesoporous architecture and larger particle size to achieve superior transverse relaxivity, a key determinant of contrast enhancement in T_2_-weighted imaging. The mesoporosity facilitates improved water accessibility to the magnetic core, thereby increasing its transverse relaxivity (r_2_) to an impressive 114.74 mM^-1^·s^-1^-substantially higher than traditional Fe_3_O_4_ nanoparticles. Furthermore, the functional coating with folic acid and chitosan endow the Fe_3_O_4_@Bio-MOF with tumor-targeting capabilities. This surface modification allows selective binding to folate receptor-overexpressing tumor cells, enhancing both imaging specificity and biocompatibility. *In vivo* MRI studies, these nanocomposites demonstrated pronounced negative signal enhancement within tumor sites shortly after administration, with signal intensity reductions of over 50% observed within one hour. This darkening effect underscores the effectiveness of Fe_3_O_4_@Bio-MOF in T_2_-weighted imaging while illustrating the potential of targeted MOF-based systems in advancing the precision of tumor diagnostics (**Figure 2B**).

Besides, recent advancements in the design and functionalization of iron-based nanoparticles, particularly SPIONs, have significantly broadened their applications in MRI, especially for tumor-targeted imaging. Through precise control of size and shape, researchers have enhanced the biodistribution and renal clearance of SPIONs, addressing challenges such as off-target accumulation and prolonged retention in non-target tissues. Surface functionalization with hydrophilic polymers, such as PEG, has further improved colloidal stability while minimizing recognition and clearance by the immune system. Importantly, the conjugation of SPIONs with tumor-targeting ligands, such as antibodies, peptides, or small molecules like folic acid, enables selective binding to cancer cell receptors, thus enhancing imaging specificity and sensitivity. For instance, in Zhang’s study, SPIONs were rationally designed for imaging HER2/neu-positive breast cancer and micrometastases (80). These nanoparticles were coated with a chitosan-grafted PEG copolymer, ensuring biocompatibility, serum stability, and minimal nonspecific interactions due to near-neutral zeta potential. Functionalized with neu-targeting antibodies, the SPIONs demonstrated high specificity for HER2/neu-expressing tumors. The MRI imaging capabilities of these SPIONs were vividly illustrated in transgenic mouse models of breast cancer. T_2_-weighted images showed significant reductions in T_2_ relaxation times 48 hours after administration of targeted SPIONs (NP-neu), highlighting their ability to accumulate at tumor sites with pronounced signal darkening, unlike nontargeted SPIONs (NP-IgG) or untreated controls. Histological staining further confirmed the presence of iron-rich nanoparticles in tumor tissues and micrometastases in the lungs and liver, as indicated by intense Prussian blue staining. These results underscore the efficacy of NP-neu in enhancing MRI contrast for both primary tumors and early micrometastatic lesions, demonstrating its potential as a precise diagnostic tool for aggressive and metastatic breast cancer. The targeted accumulation and persistent imaging contrast achieved by NP-neu exemplify the benefits of nanoparticle-based approaches in advancing the sensitivity and specificity of cancer diagnostics (**Figure 2C**).

The application of INMs in tumor imaging has been particularly impactful in preclinical studies, where their tumor-targeting capabilities have been extensively explored. Beyond localization, these nanoparticles enable dynamic imaging to monitor tumor growth, progression, and response to therapy, providing invaluable insights for clinical decision-making. As research progresses, the integration of iron-based nanoparticles into multimodal imaging platforms may further enhance their clinical relevance, solidifying their role in the future of precision oncology.

### 3.2 Photoacoustic imaging and multimodal imaging

PAI is an emerging hybrid imaging modality that combines the deep tissue penetration and high spatial resolution of ultrasound with the optical contrast provided by light absorption (81). This technique is based on the photoacoustic effect, where pulsed laser light is absorbed by tissues, generating thermoelastic expansion and ultrasound waves that can be detected and reconstructed into detailed images. The strong dependence of PAI on light absorption properties makes it highly suitable for functional imaging, such as visualizing tumor oxygenation, vascular networks, and metabolic activity (82). However, the inherent weak optical contrast of biological tissues limits its performance, necessitating the use of contrast agents to amplify the photoacoustic signal and improve imaging accuracy. INMs, with their strong optical absorption in the NIR region and magnetic properties, have shown great potential as contrast agents for PAI and in the development of multimodal imaging platforms (83).

**Figure 3:**
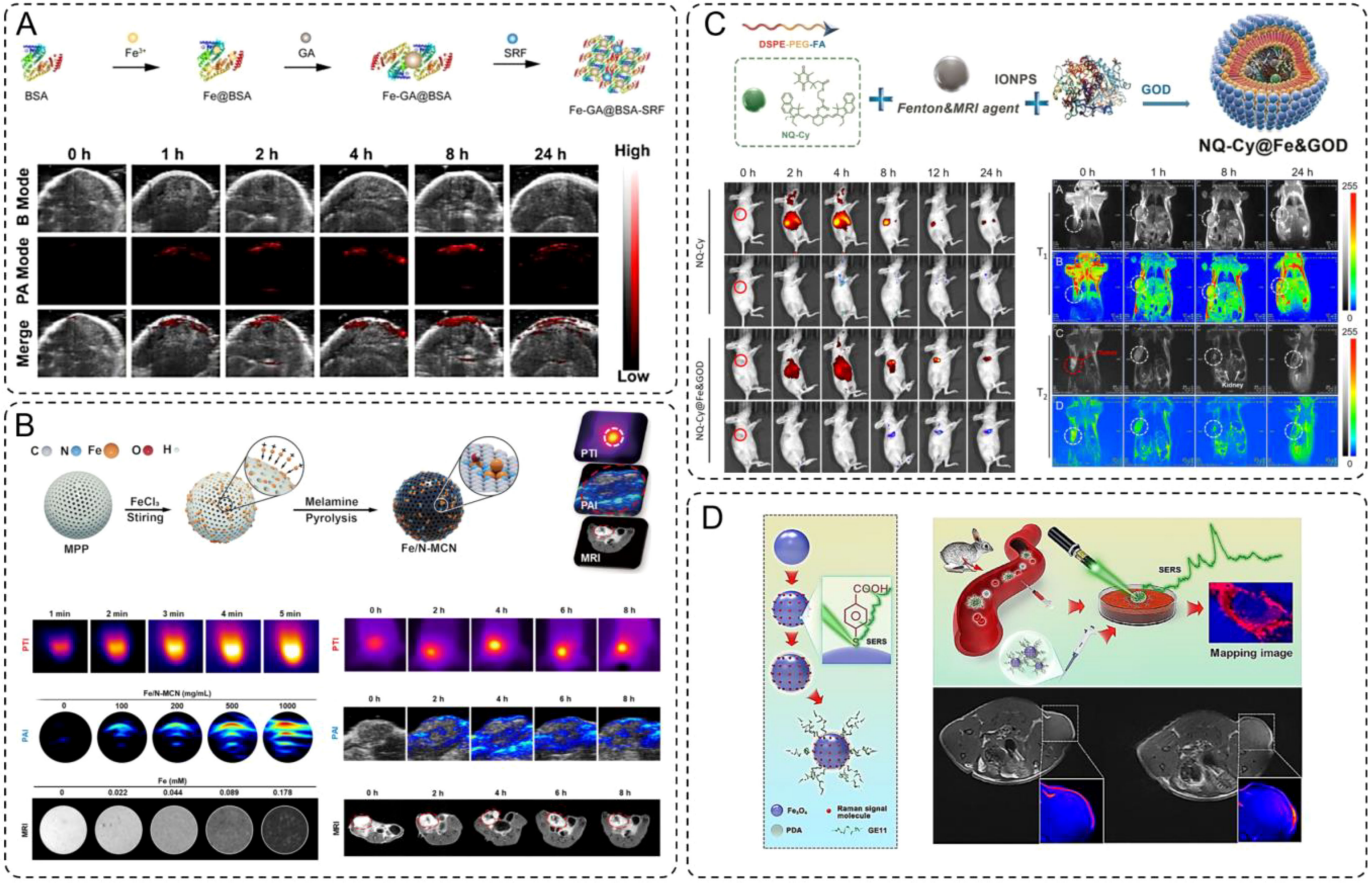
**(A)** Schematic synthesis of Fe-GA@BSA-SRF (FGB-S), and the PA images at tumor site post-injection. **(B)** Photothermal images, photoacoustic images and T2-weighted magnetic resonance images of Fe/N-MCN solution (100 μg/mL) and MCF-7 *in situ* breast cancer-bearing mice. **(C)** Schematic synthesis of the NQ-Cy@Fe&GOD. Dual-channel NIR fluorescence imaging of NQ-Cy@Fe&GOD *in vivo* tracking via intravenous injection, and mapping of the bio-distribution with MRI signal by intravenous injection. **(D)** Schematic of the synthetic preparation process for Fe_3_O_4_-AR-PDA-GE11 SERS bioprobe. And schematic diagram of SERS mapping image differentiating cancer cells in rabbit blood sample. Futher the T_1_-weighted MR images and the color-coded images of MDA-MB-231 TNB tumor-bearing nude mice before and 50 min after intravenous injection of Fe_3_O_4_-AR-PDA–GE11 bioprobes, respectively.

INMs have emerged as promising agents for PAI due to their strong optical absorption and ability to respond to tumor microenvironments. Yu et al. reported the development of Fe-GA@BSA-SRF, a polyphenol-coordinated nanomedicine constructed using ferric ions, gallic acid, and bovine serum albumin (84). This nanoparticle system features a pH-responsive design, where its stability decreases in acidic tumor environments, facilitating the controlled release of Fe^2+^ ions. These ions enhance photoacoustic contrast through efficient light absorption and conversion into acoustic signals. The nanoparticles exhibit strong and broad absorption in NIR region, peaking at 690 nm, which makes them an excellent contrast agent for PAI. *In vivo* studies demonstrated that Fe-GA@BSA-SRF accumulated effectively at the tumor site, with photoacoustic signals progressively intensifying and reaching a peak 8-hour post-injection. The signal gradually declined but remained detectable at 24-hour, indicating good retention and imaging capability. The clear tumor delineation provided by the PAI signals underscores the material’s potential for precise tumor visualization and non-invasive diagnostics. (**Figure 3A**) This innovative design not only enhances imaging depth and resolution but also establishes Fe-GA@BSA-SRF as a promising tool in advancing tumor imaging technologies, while powerful as a standalone modality, often benefits from integration with other imaging techniques to overcome limitations such as depth resolution or comprehensive anatomical visualization. The combination of PAI with other modalities, such as MRI, enables synergistic advantages, offering high spatial resolution alongside functional and molecular imaging. Recent research has focused on developing multifunctional nanomaterials capable of serving as contrast agents for such multimodal imaging systems. The work by Qin et al. highlights the development of Fe/N-MCN, a multifunctional nanoparticle system that integrates single-atom and clustered iron within nitrogen-doped mesoporous carbon nanospheres (74). This material exhibits excellent photothermal conversion efficiency and strong photoacoustic and MRI capabilities, making it a highly versatile platform for multimodal imaging applications. The unique design of Fe/N-MCN involves iron-catalyzed *in-situ* graphitization and nitrogen doping, which not only improves the structural stability of the carbon framework but also enables precise control over the distribution of iron as single atoms or clusters. This dual dispersion enhances its magnetic and optical properties, vital for efficient imaging. In imaging applications, Fe/N-MCN demonstrates exceptional performance in both PAI and MRI. The material shows broad absorption in the NIR region, facilitating strong photoacoustic signal generation and tumor site accumulation. (**Figure 3B**) PAI studies revealed significant signal intensity increases within tumors, peaking at 4 hours post-injection. Simultaneously, the incorporation of iron as a paramagnetic agent enabled effective T_2_-weighted MRI contrast, with a measured relaxivity (r_2_) of 64.64 mM^-1^·s^-1^. This combination of high-resolution PAI and MRI further underscores the potential of Fe/N-MCN as a multimodal imaging agent for precise tumor localization and monitoring.

In addition to other combinations, such as fluorescence imaging (FI) and MRI, have also garnered significant attention in multimodal imaging strategies. Building on the capabilities of fluorescence and MRI as complementary imaging modalities, Ma et al. developed a dual-modal system called NQ-Cy@Fe&GOD that combines NIR fluorescence and MRI for spatio-temporal monitoring of dose-dependent intratumoral reactions (85). This nanotheranostic system was engineered by encapsulating IONPs, glucose oxidase (GOx), and a dual-channel fluorescent probe (NQ-Cy) within a folate-modified amphiphilic polymer. (**Figure 3C**) This design not only ensures tumor-specific delivery via active targeting but also enables robust fluorescence and magnetic responses for precise imaging. The NQ-Cy@Fe&GOD system uniquely bridges the spatial resolution of MRI with the temporal sensitivity of FI. IONPs serve as T_2_-weighted MRI contrast agents, providing a linear, dose-dependent response that quantitatively maps the distribution of the nanomaterials at the tumor site with high spatial resolution. Concurrently, the NQ-Cy probe features two distinct fluorescence signals which enable real-time visualization of the therapeutic process with high temporal resolution.

So far, the integration of optical and magnetic imaging modalities has emerged as a promising approach to overcome the limitations of single-mode imaging in cancer diagnostics. While surface-enhanced Raman scattering (SERS) offers ultrasensitive molecular-level information with high spatial resolution, while MRI provides detailed anatomical insights with excellent soft-tissue contrast. Combining these two modalities enables a comprehensive imaging platform capable of precise tumor identification at both cellular and tissue levels, thus addressing challenges in early cancer detection and subtype differentiation. Lin et al. developed a dual-modal SERS–MRI nanoprobe based on ultrasmall Fe_3_O_4_ nanoparticles functionalized with alizarin red (a Raman signal molecule), polydopamine (PDA), and GE11 peptide for cancer targeting (86). This design leverages Fe_3_O_4_ unique multiple valence states to enhance SERS activity and T_1_-weighted MRI contrast. The SERS capability enabled high-resolution mapping of cancer cells, effectively distinguishing subtypes *in vitro*, while the MRI contrast demonstrated clear tumor localization *in vivo*. This system provides complementary molecular-level and deep-tissue imaging, underscoring its potential for precise cancer diagnosis and early detection (**Figure 3D**).

The unique properties of INMs make them highly effective in enhancing tumor imaging techniques. Advances in nanoparticle design, functionalization, and multimodal imaging applications have significantly improved the sensitivity, specificity, and diagnostic value of these techniques (87–89). Future research should focus on overcoming remaining challenges, such as biocompatibility and clinical translation, while leveraging AI and other computational tools to maximize the clinical potential of these materials.

## 4 Applications for iron-based nanomaterials in tumor therapy

INMs have emerged as a versatile class of agents in tumor therapy due to their unique combination of physical, chemical, and biological properties (90). Their magnetic characteristics, biocompatibility, and capacity for functionalization make them adaptable to a wide range of therapeutic strategies, from targeted drug delivery to thermal and immune-based treatments. These nanomaterials possess intrinsic magnetic properties that allow for external guidance and imaging capabilities, enabling precise localization at tumor sites (31). Moreover, their ability to participate in catalytic reactions, such as the Fenton reaction, makes them effective in generating ROS for oxidative stress-induced tumor cell death. A distinctive feature of INMs is their surface modifiability, which enables the attachment of ligands, polymers, and therapeutic agents for tumor-specific targeting and controlled release (91). For instance, their surfaces can be engineered to respond to the acidic tumor microenvironment, enhancing their selectivity and therapeutic efficacy. Additionally, their magnetic responsiveness under an AMF has paved the way for innovative approaches like magnetic hyperthermia, where localized heating induces tumor cell apoptosis while sparing healthy tissue (92). These properties allow INMs to integrate multiple therapeutic mechanisms into a single platform. Whether through immune modulation, thermal ablation, or synergistic combinations of therapies, these materials exemplify the principles of personalized and minimally invasive medicine. This multifunctionality not only enhances therapeutic precision but also reduces systemic toxicity, offering a promising avenue for next-generation cancer treatment.

### 4.1 Chemotherapy and radiotherapy

Chemotherapy and radiotherapy remain cornerstone treatments for solid tumors (93, 94). Chemotherapy involves the systemic administration of drugs to target rapidly dividing cancer cells, while radiotherapy employs high-energy radiation to damage cancer cell DNA (95). Despite their efficacy, both therapies face challenges such as drug resistance, off-target effects, and tumor hypoxia, which limit treatment outcomes (96). Integrating INMs into these therapies offers promising solutions to enhance their effectiveness.

**Figure 4:**
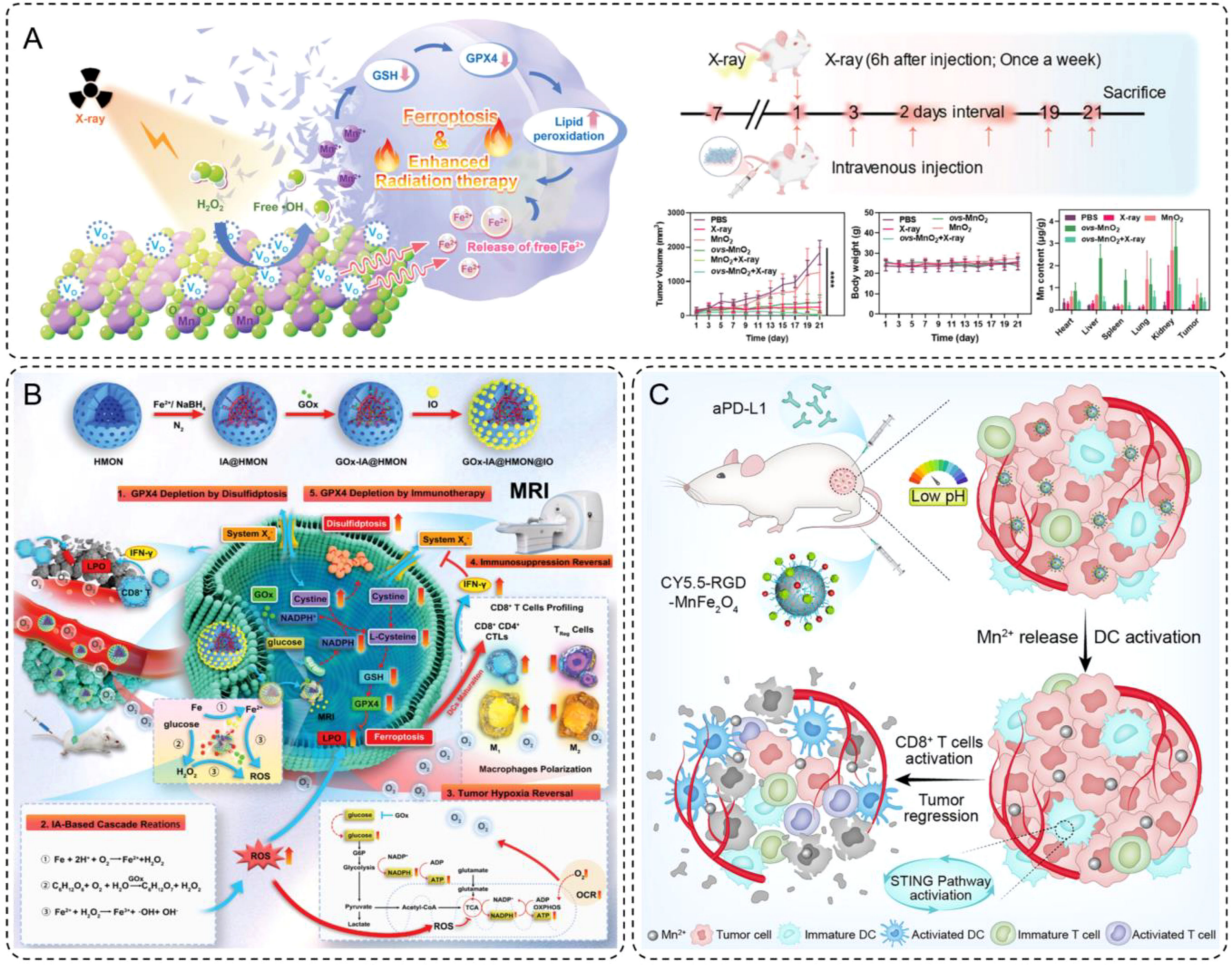
**(A)** Schematic diagram of ovs-MnO2-induced ferroptosis combined with radiotherapy. Additionally, a brief flowchart of animal experiments, and changes in tumor volume, body weight and the manganese content in various organs within 21 days of treatment. Error bars are means ± SD, n  =  5 independent repeats. **(B)** Schematic illustration for the synthesis of intelligent nanomedicine GOx-IA@HMON@IO and the mechanisms of MRI-guided tumor therapy based on synergy of ferroptosis, immunosuppression reversal, and disulfidptosis. **(C)** Schematic illustration of RGD-MnFe_2_O_4_ nanoparticles for enhancing the immunotherapeutic efficacy of aPD-L1.

Recent advances include the development of oxygen-vacancy-rich manganese dioxide (ovs-MnO_2_) nanoflowers doped with iron, which act as both ferroptosis inducers and radiosensitizers (97). Iron plays a critical role in these materials by participating in the Fenton reaction, wherein Fe^2+^ reacts with H_2_O_2_ to produce highly toxic·OH, driving oxidative stress and lipid peroxidation in tumor cells. This process induces ferroptosis, a regulated form of cell death distinct from apoptosis, which is particularly effective against resistant tumor cells. Furthermore, the release of free Fe^2+^ ions amplifies this effect, ensuring sustained ferroptotic activity. The synergy between iron-mediated ROS production and oxygen generation creates a tumor microenvironment conducive to enhanced radiation sensitivity. *In vivo* experiments demonstrated that combining ovs-MnO_2_ with radiotherapy significantly reduced tumor volumes in mouse models, while maintaining minimal systemic toxicity (**Figure 4A**).

These findings highlight the pivotal role of iron in enhancing the therapeutic potential of INMs. By integrating iron-based nanomaterials into chemotherapy and radiotherapy, we can overcome critical limitations such as hypoxia and resistance, paving the way for more effective and targeted cancer treatments.

### 4.2 Immunotherapy

INMs have shown great promise in improving the efficacy of tumor immunotherapy by reprogramming the tumor immune microenvironment and overcoming tumor-induced immunosuppression (98). These nanomaterials often possess multifunctional properties, serving both therapeutic and diagnostic purposes (99). Through carefully designed mechanisms, INMs contribute to immunotherapeutic enhancement by inducing ferroptosis-mediated immune activation and directly stimulating immune pathways, as illustrated by two recent breakthroughs.

As demonstrated by Guo et al., functionalized INMs, such as GOx-IA@HMON@IO, represent a sophisticated design aimed at catalyzing ferroptosis and inducing disulfidptosis, both of which amplify immunogenic cell death (ICD) (100). These nanomaterials were constructed with hollow mesoporous organosilica nanoparticles (HMON) as the core, loaded with GOx and *in situ*-synthesized iron atoms, and capped with ultrasmall IONPs. This intricate design enabled the release of GOx and IA in response to the elevated glutathione (GSH) levels within the tumor microenvironment. The released components facilitated a cascade of reactions, including the Fenton reaction, which generated ROS and triggered ferroptosis. (**Figure 4B**) This process depleted glutathione peroxidase 4 (GPX4) and elevated lipid peroxidation, a hallmark of ferroptosis. Additionally, the resulting ROS accumulation polarized macrophages from an immunosuppressive M2 phenotype to a pro-inflammatory M1 phenotype, promoting the activation of cytotoxic T lymphocytes (CTLs). This macrophage polarization, coupled with the release of tumor antigens during ferroptosis and disulfidptosis, led to enhanced CD8+ T cell infiltration and a reversal of the immunosuppressive tumor microenvironment. This cascade of evidence illustrates the critical role of INM design in achieving synergistic therapeutic effects through ferroptosis and immunomodulation.

Building on this principle, Shi et al. developed manganese-ferrite nanoparticles (MnFe_2_O_4_) functionalized with RGD-targeting ligands, demonstrating another innovative approach to immunotherapeutic enhancement (101). The MnFe_2_O_4_ nanoparticles were engineered with a core-shell structure, where the manganese ferrite core provided MRI capabilities and the RGD peptides on the shell enabled targeted delivery to integrin-overexpressing tumor cells. (**Figure 4C**) Once internalized into the acidic tumor microenvironment, these nanoparticles released Mn^2+^ ions in a controlled manner. These ions activated the stimulator of interferon genes (STING) signaling pathway, a critical axis in adaptive immunity. By promoting TBK1 phosphorylation and upregulating PD-L1 expression, the STING activation facilitated a more immunogenic tumor microenvironment. When combined with anti-PD-L1 antibodies, the MnFe_2_O_4_ nanoparticles significantly enhanced the infiltration of both CD8+ and CD4+ T cells into tumors, amplifying the immune response. Furthermore, the careful design of these nanoparticles ensured high tumor specificity and retention, minimizing off-target effects while providing a platform for both immune activation and imaging.

Together, these studies highlight the transformative potential of INMs in immunotherapy. By integrating immunomodulatory effects with advanced material design and imaging capabilities, these nanomaterials address key challenges in tumor immunotherapy. Their ability to reprogram the tumor immune microenvironment, enhance antigen presentation, and synergize with existing therapies underscores their value as powerful tools for next-generation cancer treatment. These advances pave the way for the broader application of INMs in personalized and precision medicine, bridging the gap between therapeutic efficacy and diagnostic precision.

### 4.3 Magnetic hyperthermia therapy

Thermal therapies have long been recognized as a promising strategy for cancer treatment, utilizing localized heat to ablate tumor cells or modulate their biological behavior (102). Among these, magnetic hyperthermia has gained attention due to its ability to provide non-invasive (103), controlled, deep tissue heating by leveraging magnetic nanoparticles under an AMF (35). Unlike conventional thermal ablation methods, magnetic hyperthermia minimizes collateral damage by targeting tumor tissues specifically, offering precision in therapy delivery (104). Recent advances in material design have further enhanced the efficacy and safety of magnetic hyperthermia, allowing for minimally invasive delivery and even integration with immune modulation.

**Figure 5:**
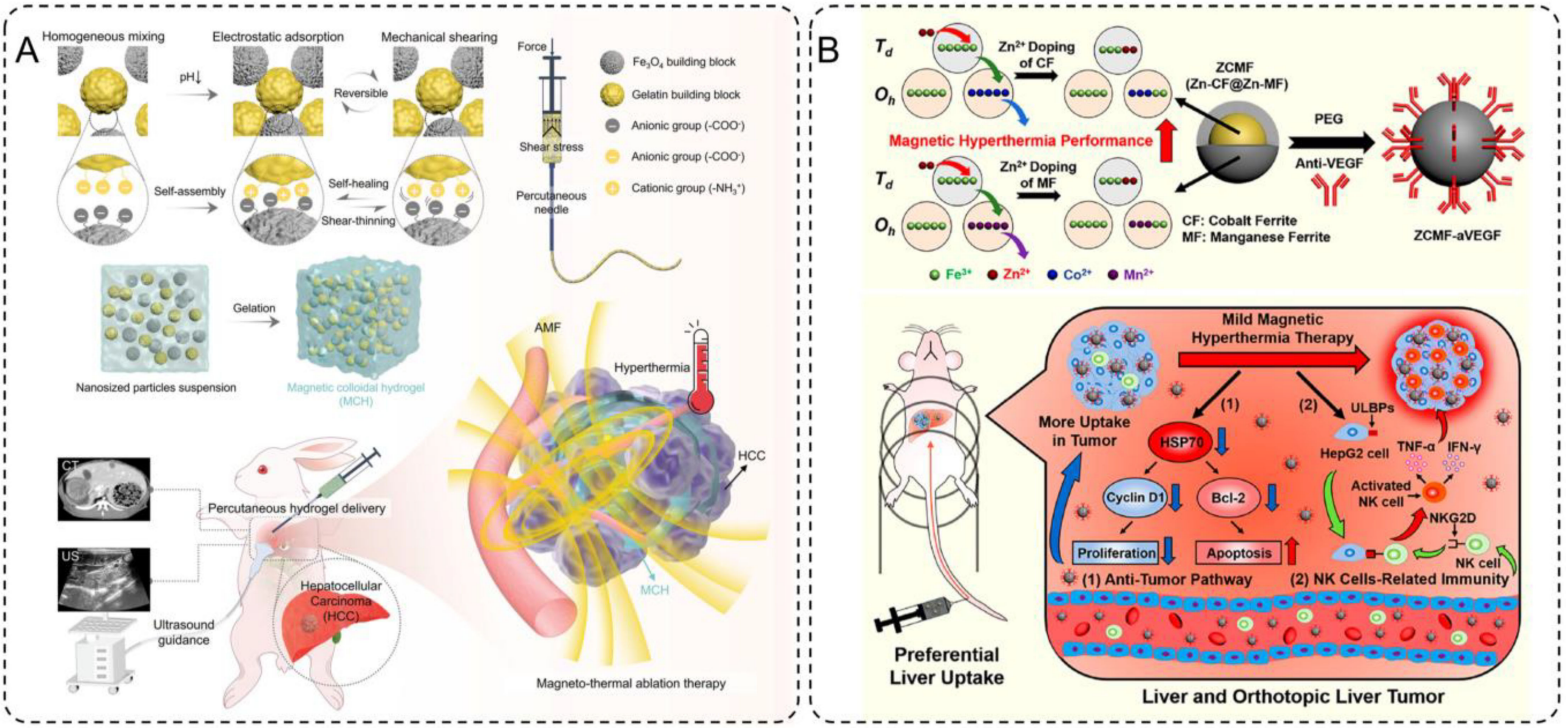
**(A)** Design and preparation of the injectable hydrogel ablation agent for ultrasound-guided magnetic hyperthermia ablation of HCC. **(B)** Schematics of magnetism enhancements, mild magnetic hyperthermia, and antitumor immunity induction for liver cancer therapy.

One notable development in this field is the magnetic colloidal hydrogel (MCH) designed by Wu et al., which combines Fe_3_O_4_ nanoparticles with gelatin building blocks for the treatment of hepatocellular carcinoma (HCC) (105). This hydrogel exhibits self-healing, shear-thinning, and gelation properties, enabling precise percutaneous injection under ultrasound guidance without material leakage. Once delivered to the tumor site, the Fe_3_O_4_ nanoparticles generate localized heat under AMF exposure, effectively ablating tumor tissues while sparing healthy ones. (**Figure 5A**) This approach not only reduces recovery times compared to conventional methods but also ensures precise treatment delivery. In preclinical models, the MCH demonstrated significant tumor burden reduction while maintaining excellent biocompatibility, highlighting its potential as a minimally invasive therapeutic platform.

Building on this concept of controlled heating, Pan et al. explored the use of Zn-CoFe_2_O_4_@Zn-MnFe_2_O_4_ nanoparticles (ZCMF) for mild magnetic hyperthermia at temperatures of 43-44°C (106). By leveraging a core-shell structure and Zn-doping, these nanoparticles achieve enhanced magnetic hyperthermia performance with precise temperature control. This mild hyperthermia not only directly inhibits tumor growth by suppressing heat shock protein 70 (HSP70), a key factor in tumor cell survival, but also activates natural killer (NK) cells by upregulating ligands like ULBPs on tumor cells. (**Figure 5B**) This dual mechanism enables a synergistic therapeutic effect, combining localized tumor ablation with systemic immune activation. Furthermore, the natural uptake of ZCMF nanoparticles by the liver through the mononuclear phagocyte system enhances their accumulation at the target site, providing an effective solution for liver cancer treatment. Preclinical studies demonstrated significant tumor regression and immune activation, showcasing the potential of mild magnetic hyperthermia as a comprehensive therapeutic strategy.

Together, these advancements highlight the significant potential of INMs in magnetic hyperthermia for cancer treatment. Their unique properties, including magnetic responsiveness, biocompatibility, and the ability to be engineered for precise delivery and controlled heating, make them ideal candidates for thermal therapies. By generating localized heat under an AMF, these materials enable targeted tumor ablation with minimal damage to surrounding healthy tissues. Furthermore, innovations in material design, such as hydrogels and core-shell nanoparticles, have expanded the applicability of magnetic hyperthermia, improving efficiency and safety. These developments underline the promise of INMs as powerful tools in advancing cancer treatment through minimally invasive and precise thermal therapies.

### 4.4 Harnessing synergy in combined therapeutics

**Figure 6:**
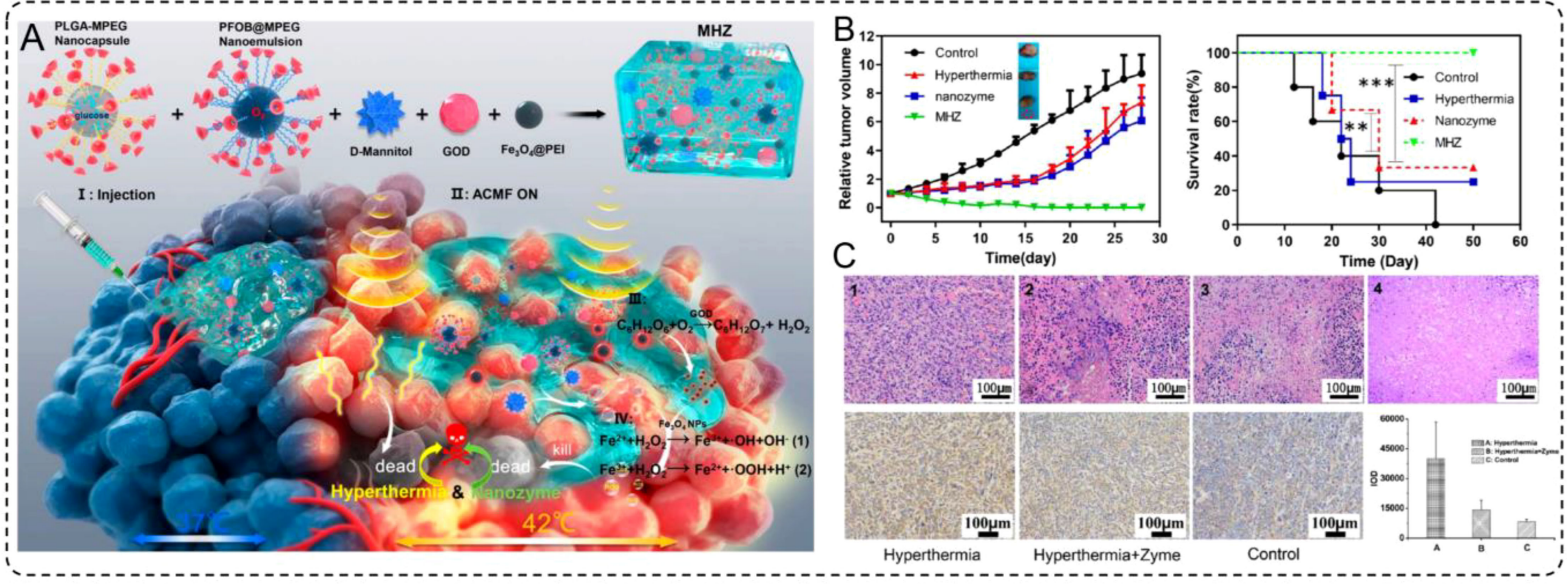
**(A)** Schematic illustration for the microstructure and application of MHZ, and synthetic procedure and composition for MHZ. **(B)** Scheme of synergistic mechanism of MHZ on the generation of hyperthermia and ROS for cancer therapy. **(B)** The relative tumor volumes of different groups after treatment. **(C)** Long-term survival rates of mice bearing 4T1 tumors after several treatment processes as indicated.

The integration of multiple therapeutic mechanisms within a single platform has emerged as a potent strategy to overcome the limitations of standalone cancer therapies (107, 108). By combining hyperthermia with catalytic therapy, INMs can effectively address tumor resistance mechanisms, enhance oxidative stress, and amplify tumor cell death while minimizing damage to surrounding tissues. One notable example illustrating this approach is the development of magnetic hydrogel nanozymes (MHZ), which leverage the unique properties of magnetic nanoparticles and enzyme-like activity to synergistically enhance therapeutic efficacy. Wu et al. (2019) developed an injectable MHZ system by combining Fe_3_O_4_ nanoparticles, GOx, and D-mannitol within a shear-thinning hydrogel. This design enables precise tumor delivery through percutaneous injections and stable retention in the tumor microenvironment. Under an AMF, Fe_3_O_4_ nanoparticles generate mild hyperthermia (~42°C) while activating GOx to catalyze the production of ROS through a Fenton-like reaction, inducing oxidative damage and subsequent tumor cell death (**Figure 6A**).

In preclinical models, MHZ demonstrated remarkable tumor suppression (**Figure 6B**) and prolonged survival rates (**Figure 6C**) compared to hyperthermia or catalytic therapy alone. The MHZ-treated group exhibited significantly reduced tumor volume, with histological analysis revealing extensive tumor necrosis but minimal damage to adjacent healthy tissues. These findings underscore the potential of MHZ as a powerful tool for synergistic cancer therapy that combines precise thermal control from hyperthermia with potent oxidative damage induced by catalytic therapy. This approach not only enhances therapeutic efficacy but also represents a minimally invasive and highly targeted treatment modality for challenging types of tumors. Therefore, the synergistic effects of INMs not only enhance oxidative stress but also inhibit the expression of heat shock proteins, resulting in a significant weakening of tumor defenses. This approach demonstrates the versatility of INMs in combining thermal and catalytic effects, showcasing their potential for precise, minimally invasive, and highly effective cancer treatments.

## 5 Integration of iron-based nanomaterials with artificial intelligence in tumor diagnosis and therapy

The advent of AI has revolutionized tumor diagnosis and treatment by enabling data-driven, precise, and predictive approaches. Meanwhile, INMs, with their unique physicochemical properties, have shown remarkable promise in enhancing imaging sensitivity and therapeutic efficacy. Integrating these advanced nanomaterials with AI presents an opportunity to develop innovative strategies for personalized cancer care (109, 110). This section explores the current progress, challenges, and future directions of combining INMs with AI in tumor diagnosis and therapy.

### 5.1 The role of AI in enhancing INM-based applications

**Figure 7:**
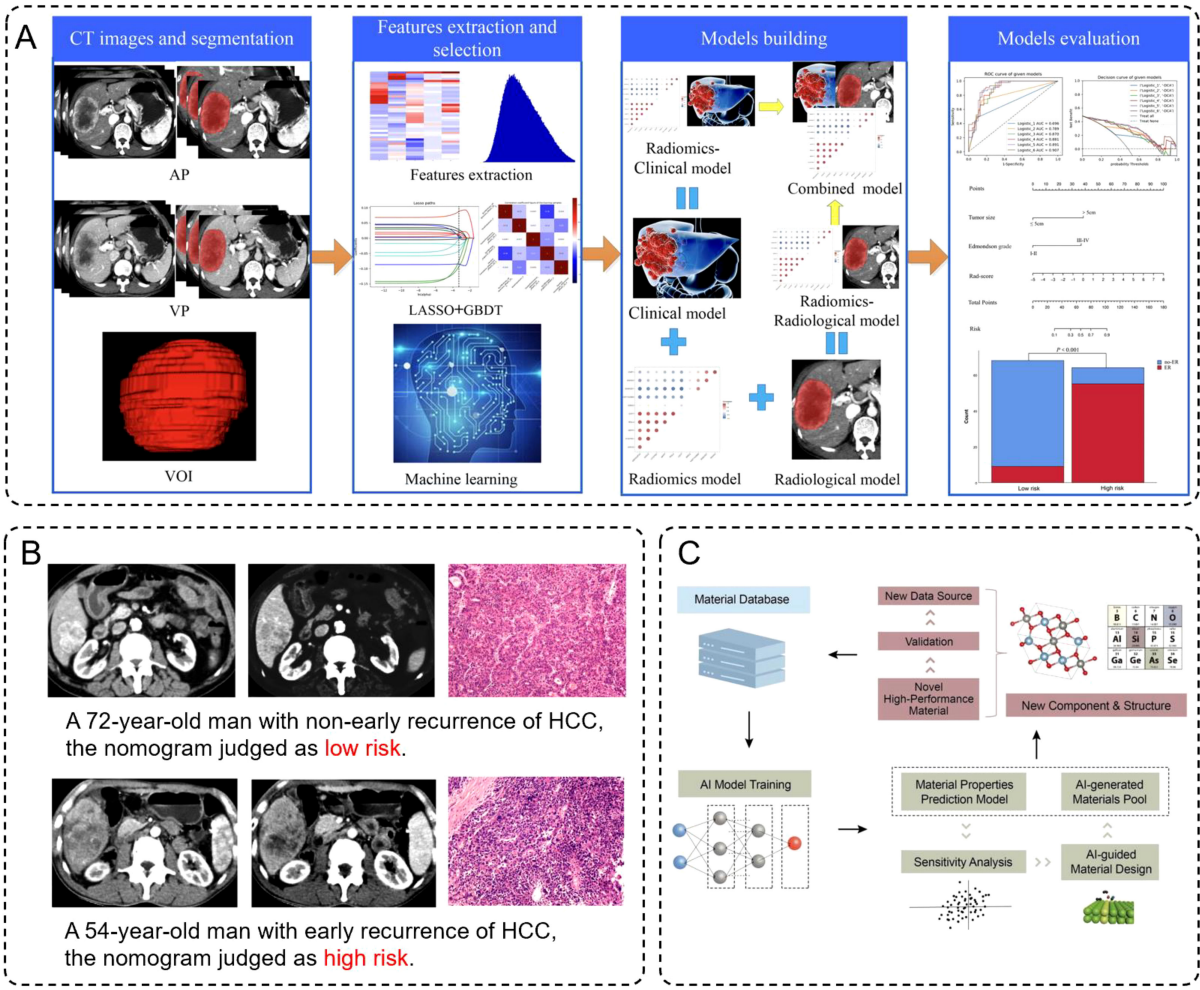
**(A)** Schematic diagram of construction and evaluation of models. **(B)** Preoperative contra-enhanced CT images and pathological pictures of two cases with HCC. **(C)** Framework for training models, in which learning from the existing database, AI technology can understand the material’s properties based on the representative material’s descriptor.

Artificial intelligence, particularly machine learning and deep learning techniques has demonstrated significant capabilities in analyzing complex medical imaging data (111), making it a valuable complement to iron-based nanomaterial-enhanced modalities. Radiomics, a core application of AI, involves extracting large sets of quantitative features from imaging data to characterize tumor phenotypes and predict clinical outcomes (112–115). By leveraging AI algorithms to process CT and MRI imaging data, large quantities of quantitative features can be extracted for disease prediction. For example, Wu et al. developed a CT-based radiomics nomogram, significantly improving the stratification and prediction of early recurrence risk in hepatocellular carcinoma (HCC) patients post-surgery (116). Their workflow demonstrated the complete process from CT image segmentation, feature extraction, and model construction to model evaluation (**Figure 7A**), which highlighted how the integration of radiomics and clinical data enhances the predictive accuracy for early HCC recurrence. Furthermore, patient-specific cases validated the model’s effectiveness by comparing risk assessments with actual recurrence outcomes (**Figure 7B**). Similarly, Kim et al. utilized MRI-based radiomics to predict both early and late recurrence of HCC, showing performance comparable to traditional clinical-pathological models (117).

The integration of omics data with imaging techniques provides another pathway to enhance the diagnostic and therapeutic utility of INMs. Multi-omics data, including genomics, transcriptomics, and metabolomics, can be combined with radiomics to create a comprehensive understanding of the tumor microenvironment (118, 119). For example, incorporating genetic profiles of iron metabolism or oxidative stress pathways could provide insights into the effectiveness of iron-based therapies like ferroptosis. AI models trained on such multimodal datasets can identify predictive biomarkers and therapeutic targets, enabling more personalized and effective treatment strategies (120, 121).

Besides, deep learning (DL) has demonstrated tremendous potential in the design and optimization of nanoparticles. By analyzing experimental and computational data, AI can identify optimal nanoparticle sizes, shapes, and surface modifications to enhance their imaging and therapeutic properties (122, 123). For instance, Witten et al. utilized deep learning models to optimize the RNA delivery efficiency of lipid nanoparticles, significantly advancing gene therapy (124). Moreover, Han et al. developed an AI-guided framework for materials design, where machine learning models predicted thermoelectric (TE) properties such as electrical conductivity, thermal conductivity, and Seebeck coefficients. These predictions were validated through density functional theory (DFT), leading to the discovery of multiple high-performance TE materials. This framework showcased a workflow for precise property prediction and the rapid identification of new materials through descriptor design and sensitivity analysis (**Figure 7C**). Although Han et al.’s work primarily focuses on TE materials, their AI framework offers valuable insights into INM design and optimization. By incorporating similar descriptor designs and sensitivity analyses, we can more efficiently predict and regulate the surface modifications, size distributions, and physicochemical properties of INMs, achieving superior performance in imaging and therapeutic applications. This cross-disciplinary innovation not only provides a new research avenue for nanomaterials science but also lays a foundation for advancing intelligent medicine.

### 5.2 Challenges in combining AI and INMs and future directions

Although significant advances have been achieved in both AI-assisted radiomics and deep learning approaches for the design of nanoparticles, their applications to iron-based nanomaterials remain in their infancy. Iron-based nanomaterials possess superior magnetic properties and excellent chemical stability; thus, they have widely been used in both MRI and cancer therapy. However, the heterogeneity of clinical and omics data, the absence of standardized data processing, and lack of sufficient clinical validation of AI models are major challenges to wider applications. Moreover, how to integrate these multimodal data into AI models for comprehensive material properties is still a big challenge.

In the future, overcoming these challenges will enable AI-driven iron-based nanomaterial design to realize personalized tumor imaging and precision therapy. This development is expected to significantly improve the adaptability of materials to complicated disease environments and further accelerate the development and clinical translation of novel functionalized nanomaterials. As more data accumulate and AI algorithms are further optimized, research into the intersection between AI and iron-based nanomaterials will be a key direction in personalized cancer therapy and open up new avenues for intelligent medical development.

## 6 Conclusion

INMs have indeed shown great potential in the advancement of tumor imaging and therapy by acting as multifunctional platforms for precision oncology. Their unique magnetic properties, excellent biocompatibility, and capacity to integrate diagnostic and therapeutic modalities make them highly valued tools in addressing the limitations of traditional approaches to the management of cancer. From the enhancement of imaging sensitivity and specificity in applications within MRI and PAI to allowing for precise, less-invasive treatments like PTT, immunotherapy, and magnetic hyperthermia, such materials have so far contributed to cancer diagnostics and therapeutics. Beyond their established applications, the integration of INMs with advanced technologies such as AI, radiomics, and immunotherapy is paving the way toward the development of next-generation cancer treatment strategies. These innovations have the potential to revolutionize patient care by offering real-time, personalized, and adaptive solutions that optimize treatment efficacy while minimizing side effects.

However, several challenges need to be overcome before INMs can see complete clinical translation, including the enhancement of synergistic interactions between imaging and therapeutic functionalities, long-term safety and biodegradability, and issues with large-scale production and regulatory approval. The success of ferumoxytol serves as a template to address such complexities and fully realize the transformative potential of INMs. Overcoming these challenges will require an interdisciplinary approach. In the future, collaboration between researchers, clinicians, and industrial partners will also be essential to rapid advancements in this area. By uniting efforts across disciplines, we can unlock the full potential of INMs to transform cancer diagnosis and treatment, ultimately improving outcomes for patients worldwide.
